# Optimizing TB Bacteria Detection Efficiency: Utilizing RetinaNet-Based Preprocessing Techniques for Small Image Patch Classification

**DOI:** 10.1155/ijbi/3559598

**Published:** 2025-10-05

**Authors:** Shwetha V., Barnini Banerjee, Vijaya Laxmi, Priya Kamath

**Affiliations:** ^1^Department of Electrical and Electronics Engineering, Manipal Institute of Technology, Manipal, Manipal Academy of Higher Education, Manipal, Karnataka, India; ^2^Department of Microbiology, Kasturba Medical College, Manipal, Manipal Academy of Higher Education, Manipal, Karnataka, India; ^3^Department of Computer Science and Engineering, Manipal Institute of Technology, Manipal, Manipal Academy of Higher Education, Manipal, Karnataka, India

**Keywords:** biomedical imaging, computer-aided diagnosis, feature extraction, image patch analysis, image processing, machine learning, pre-processing techniques, RetinaNet, small image patch classification, TB bacteria detection

## Abstract

Tuberculosis (TB), caused by *Mycobacterium tuberculosis*, is a re-emerging disease that necessitates early and accurate detection. While Ziehl–Neelsen (ZN) staining is effective in highlighting bacterial morphology, automation significantly accelerates the diagnostic workflow. However, detecting TB bacilli—which are typically much smaller than white blood cells (WBCs)—in stained images remains a considerable challenge. This study leverages the ZNSM-iDB dataset, which comprises approximately 2000 publicly available images captured using different staining methods. Notably, 800 images are fully stained with the ZN technique. We propose a novel two-stage pipeline where a RetinaNet-based object detection model functions as a preprocessing step to localize and isolate TB bacilli and WBCs from ZN-stained images. To address the challenges posed by low spatial resolution and background interference, the RetinaNet model is enhanced with dilated convolutional layers to improve fine-grained feature extraction. This approach not only facilitates accurate detection of small objects but also achieves an average precision (AP) of 0.94 for WBCs and 0.97 for TB bacilli. Following detection, a patch-based convolutional neural network (CNN) classifier is employed to classify the extracted regions. The proposed CNN model achieves a remarkable classification accuracy of 93%, outperforming other traditional CNN architectures. This framework demonstrates a robust and scalable solution for automated TB screening using ZN-stained microscopy images.

## 1. Introduction

Tuberculosis (TB), caused by *Mycobacterium tuberculosis*, is a re-emerging global health challenge due to drug-resistant strains [1]. Early detection is crucial to controlling its spread and improving patient outcomes. Ziehl–Neelsen (ZN) staining has traditionally played a pivotal role in identifying *Mycobacterium tuberculosis* in sputum samples because of its ability to highlight acid-fast bacilli (AFB) [2]. However, manual microscopy is time-consuming, labor-intensive, and prone to errors due to human fatigue [3].

Recent advancements in machine learning, particularly in object detection, have introduced the potential for automating TB diagnostics. Convolutional neural networks (CNNs) are increasingly being applied in medical imaging tasks [4], yet detecting and classifying small objects, such as TB bacilli, in stained images poses a significant challenge due to their small size and the noisy backgrounds typically found in these samples [5, 6].

Traditional object detectors, such as single-shot detectors (SSDs) [7] and YOLO [8], have proven to be effective in various applications but struggle when tasked with detecting small objects. In ZN-stained images, TB bacilli are often smaller than white blood cells (WBCs), exacerbating the issue. To address these challenges, RetinaNet, with its feature pyramid network (FPN) and focal loss, has emerged as a promising solution for small object detection [9]. Nevertheless, standard RetinaNet configurations may not capture sufficient detail for fine structures like TB bacilli.

To enhance detection performance, we propose modifying RetinaNet by incorporating dilated convolutional layers, which allow for finer feature extraction at multiple scales [10]. This adaptation improves the model's ability to isolate and detect TB bacteria and WBCs in ZN-stained images. As a preprocessing step, this enhanced RetinaNet effectively identifies regions of interest, reducing noise and focusing attention on smaller objects [11].

Although numerous studies have focused on deep learning–based detection of TB bacilli from ZN-stained images, several critical gaps remain unaddressed [12–14]. Current object detection models, such as SSD and YOLO, show limitations in detecting smaller objects, particularly in medical imaging where precision is crucial [7, 15]. Furthermore, most CNN-based classifiers do not adequately handle the challenge posed by low-resolution and noisy medical images where both background interference and object size variability present significant hurdles [16, 17]. Our approach addresses these gaps by focusing on the following aspects.

Efficient detection and classification of TB bacilli in ZN-stained images remains a significant challenge due to the small size of the bacilli and their often low contrast against noisy backgrounds. Most current approaches lack efficient preprocessing techniques tailored for small object detection. Traditional models struggle to isolate small regions of interest (ROIs) in medical images, particularly where the target objects are significantly smaller than other elements like WBCs. To address this, we introduce a RetinaNet-based object detection model enhanced with dilated convolutions, which efficiently isolates small ROIs and reduces noise, improving downstream classification accuracy [9, 18].

TB bacilli pose further challenges in classification due to their small size and low resolution in ZN-stained patches. Conventional CNN classifiers fail to handle small patches effectively, particularly those in the range of 30 × 30 to 120 × 120 pixels. Our proposed CNN-based classifier is specifically designed to improve the classification performance on such small input patches, outperforming traditional models [19]. Additionally, class imbalance and background interference are persistent issues in TB detection. Many existing methods suffer from performance degradation in the presence of noisy backgrounds and skewed class distributions between TB bacilli and larger elements like WBCs. To tackle these issues, we leverage RetinaNet's focal loss to address class imbalance while dilated convolutions reduce background interference, allowing the CNN classifier to focus on more relevant features in the ROIs [9, 19].

Comparative analysis with existing classifiers, such as ResNet [20] and DenseNet [21], demonstrates the robustness of our approach. Our model achieves a classification accuracy of 93.12%, outperforming these popular architectures and highlighting the effectiveness of the proposed methods in detecting small objects in noisy medical images.

The key contributions of this work can be summarized as follows:

• A novel preprocessing approach using an enhanced RetinaNet-based object detection model to isolate small ROIs, specifically focusing on TB bacilli and WBCs in ZN-stained images.
• Introduction of a CNN classifier optimized for classifying small images (30 × 30 to 120 × 120 pixels) that achieves 93.12% accuracy, outperforming state-of-the-art CNN classifiers.
• Effective handling of class imbalance and background noise through the use of focal loss and dilated convolutions in the RetinaNet model, leading to significant improvements in TB detection from ZN-stained samples.
• Comprehensive comparative analysis with other CNN-based classifiers, highlighting the superior performance of the proposed methodology.

## 2. Related Works

The literature on TB bacteria detection has evolved from basic machine learning techniques to sophisticated deep learning models, particularly CNNs. Object detection models have progressively incorporated advanced algorithms and hardware solutions to enhance accuracy and efficiency. Meanwhile, CNN-based classification has expanded its capabilities through transfer learning, synthetic data generation, and architecture optimization. The focus on small image classification using CNNs highlights the ongoing efforts to address unique challenges associated with limited data, emphasizing the need for models that can accurately interpret and classify small-scale features. Together, these advancements demonstrate a robust and comprehensive approach to improving TB bacteria detection, described in the following section.

### 2.1. Object Detection Models for Detection of Bacteria

Research in automated TB bacteria detection has explored various object detection models that aim to identify bacteria accurately within images. For instance, Forero et al. [22] developed an autofocus algorithm that achieved a specificity of 93.5%, leveraging specific features of TB bacteria to improve detection and classification accuracy. Similarly, Shi et al. [23] utilized HSV color space in a machine learning model to detect TB bacteria, effectively reducing noise through threshold filters. Morphological features such as size, shape, and stain intensity have been pivotal in identifying rod-shaped bacteria across several studies [24]. Ladgham et al. [24] introduced a hardware-based system for fluorescent TB bacteria detection, employing steps such as RGB to grayscale conversion and threshold filtering. This system, implemented on a field programmable gate array (FPGA), utilized only 1% of its resources and 691 configurable logic blocks (CLBs). More recently, Osman et al. [25] proposed using extreme machine learning approaches for TB detection, complemented by adaptive filtering techniques [26]. Zhang et al. [27] enhanced small object detection with a modified YOLO model, optimizing feature map resolution to improve accuracy for detecting small TB bacteria. Zhou et al. [28] further refined detection methodologies by integrating a FPN into an enhanced faster R-CNN model, which better accommodates multiscale bacteria detection tasks. Additionally, Wang et al. [29] proposed a novel multiscale attention network that dynamically adjusts focus on bacteria of varying sizes within an image, further enhancing detection accuracy.

### 2.2. CNN-Based Classification

CNNs have emerged as a powerful tool for TB bacteria detection due to their superior feature extraction capabilities and robustness in handling diverse data types. Transfer learning with models such as AlexNet [30] and ResNet-50 has shown promising results.

Ref. [20] has led to significant improvements in classification accuracy. El-Melegy et al. [31] utilized faster R-CNN to refine TB bacteria classification, achieving notable advancements in detection rates. A segmentation model by Panigrahi et al. [32] was developed to automatically segment complex bacterial communities, effectively identifying individual bacteria within mixed cultures. Xiong et al. [33] reported an AI-assisted approach for detecting acid-fast–stained TB bacilli with a specificity of 83.65%.

Recent studies have focused on enhancing CNN architectures for TB bacteria detection. Li et al. [34] explored the use of generative adversarial networks (GANs) to produce synthetic data, thereby improving the training process of CNN models. Additionally, Li et al. [35] designed a lightweight CNN architecture optimized for deployment on mobile devices, demonstrating high accuracy and low computational cost, which is crucial for TB detection in low-resource settings.

Furthermore, advances in deep neural network pruning [36] have enabled more efficient CNN models, reducing computational requirements while maintaining high detection performance.

### 2.3. Small Image Classification Using CNNs

Classifying small images, particularly in the context of TB bacteria detection, presents unique challenges due to the limited pixel information available. CNN-based models have been adapted to address these challenges by incorporating specialized architectures that focus on fine-grained feature extraction. For instance, Chao et al. [37] proposed a deep residual shrinkage network that effectively reduces noise while enhancing the important features in small images of bacteria. Similarly, Anwar et al. [38] introduced an optimized shallow CNN model specifically designed for small image inputs, achieving improved accuracy by minimizing the model's depth to prevent overfitting on limited data.

In another study, Zhang et al. [39] utilized a capsule network to retain spatial hierarchies, which are often lost in traditional CNNs when processing small images. This approach has shown promising results in accurately classifying TB bacteria by preserving the relationships between features across different scales.

Furthermore, enhancements in attention mechanisms have allowed CNNs to dynamically adjust focus on the most relevant parts of an image, significantly improving classification accuracy for small TB bacteria images [40].

## 3. Methodology

The input images, stained using the ZN staining technique, are characterized by dimensions of 1800 × 1500 pixels. Bacilli and WBCs are extracted from these high-resolution images using a modified RetinaNet model specifically designed for small object detection. This model employs anchor boxes to identify and isolate ROIs, ensuring that even the smallest features within the images are accurately captured.

**Figure 1:**
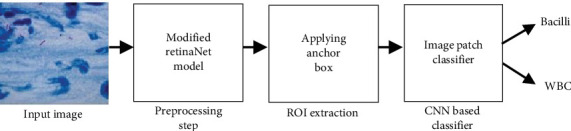
Overall proposed methodology for TB bacteria detection using RetinaNet and CNN classifiers.

Following the ROI extraction, a CNN-based classification framework is implemented to analyze the image patches further. These patches, which vary in size from 26 × 26 to 128 × 128 pixels, are classified into two primary categories: TB bacteria and WBCs. This multistage approach leverages object detection and deep learning's strengths, ensuring high precision in distinguishing between these categories. The detailed methodology employed in this study is depicted in Figure 1, providing a comprehensive overview of the process from image acquisition to classification.

### 3.1. Dataset

**Figure 2:**
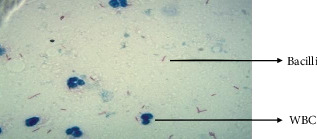
Sample images used in the study.

The ZNSM-iDB database comprises approximately 2000 publicly accessible images obtained through various staining techniques [41]. Among these, 800 images are fully stained using the ZN staining method and captured at a 25× magnification.  Figure 2 highlights the visibility of WBC and bacteria in these ZN-stained images. A detailed overview of the dataset utilized in this study is provided in Figure 2.

### 3.2. ROI Extraction Using Modified RetinaNet Model

In our study, we utilized the Keras implementation of RetinaNet [9] as the baseline architecture and introduced specific modifications aimed at enhancing the detection performance for small objects. Drawing inspiration from prior research [20], we made significant adjustments to the ResNet-50 backbone [20], which consists of four convolutional blocks. Initially, we leveraged the last three convolutional blocks to construct the feature pyramid, resulting in spatial scales of 8×, 16×, and 32×, respectively.

To better localize small objects and mitigate information loss within deep feature maps, we replaced blocks that produced output feature maps with a downsampling rate exceeding 8× with newly designed bottleneck blocks. Additionally, we incorporated dilated convolutional layers, as recommended by [10], to expand the spatial resolution of feature maps to 64× and 128×, thereby enhancing the detection of larger objects.

The feature pyramids were constructed with lateral connections from Stages 3 to 5 (p3–p5), while the downsampling pyramids (p6 and p7), typically focused on large object detection, were excluded. To further refine feature fusion, we adopted a novel approach inspired by recent developments in HRNet [42]. This involved adding an extra convolutional block after the dilated bottleneck layer, which was designed to extract features with enhanced semantic relevance.

**Figure 3:**
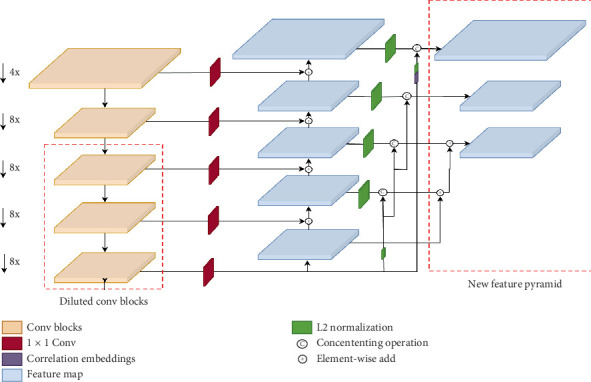
Modified RetinaNet model used in the study.

The subsequent feature pyramids (P1–P4) were guided by direct, high-level semantic information, allowing for more effective integration of low-level features with the output from the deepest layers. This innovative method capitalizes on high-level guidance from the deeper layers of the network to improve classification accuracy, particularly for small objects that share similar characteristics across different classes. The proposed modifications are illustrated in Figure 3.

### 3.3. Image Patch Classifier

The model also includes additional structural refinements, such as progressively increasing the number of filters across convolutional layers to capture hierarchical features effectively. In the initial layer, a smaller number of filters are applied to detect low-level features, while deeper layers utilize larger filter sets to extract more abstract, high-level patterns relevant to TB detection.

To further improve the model performance, the optimizer employed is Adam, which dynamically adjusts the learning rate during training, enhancing stability and convergence. The loss function chosen is categorical cross-entropy, suited for the multiclass classification task. Furthermore, early stopping criteria are integrated, and validation loss is monitored to prevent overfitting by halting training once performance plateaus.

**Figure 4:**
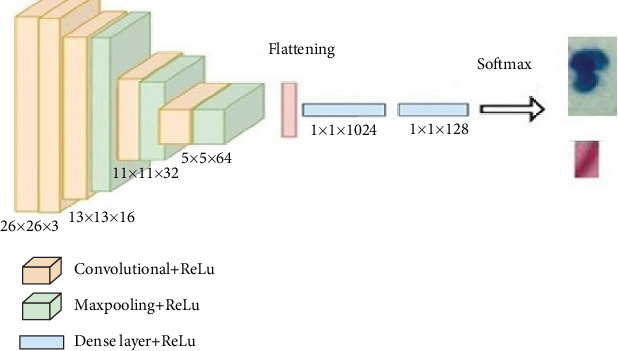
The overall architecture in the study.

Finally, the model's architecture is designed to handle the relatively small input sizes efficiently, ensuring that the network complexity aligns with the dataset's characteristics and optimizes computational resources without sacrificing accuracy. The overall architecture is shown in Figure 4.

The proposed CNN model for the detection and classification of TB bacteria is detailed in Table 1.

### 3.4. Implementation Details

In this section, we have discussed the implementation of RetinaNet and the CNN classifier, which will be detailed further in the subsequent section.

#### 3.4.1. RetinaNet Object Detection and Extraction

The detection model was implemented on the PyTorch platform. Subsequently, 1200 subimages were extracted from 800 full images, with 850 images allocated to the training set and 350 to the test set. Each image underwent preprocessing, including resizing to 512 × 512 pixels and normalization of grey values to [0, 1] by dividing by 256. Features were extracted from ZN images using the RetinaNet model with ResNet, and the FPN generated the final features. The model was trained with an IOU threshold of 0.5, a batch size of 16, and a kernel size of 3. ReLU activation functions were employed in the constitutional layers of all deep networks.

A notable aspect of this process was extracting rectangular boundary boxes for ROI, specifically targeting TB bacteria and WBCs. The RetinaNet model was specifically adapted to detect these rectangular boundaries, ensuring precise localization of TB bacteria and WBCs within the images.

The RetinaNet model employs focal loss to address the class imbalance inherent in object detection tasks [43]. Focal loss modifies the standard cross-entropy loss by downweighting the loss assigned to well-classified examples, allowing the model to focus more on hard-to-classify examples [44]. This approach helps improve detection performance, especially when the number of foreground objects (TB bacteria or WBCs) is small compared to the number of background examples.

Focal loss is defined as follows:

(1) $$\begin{array}{c} \text{FL}\left(p_{t}\right)=-\alpha_{t}\left(1-p_{t}\right)\gamma \;\log\left(p_{t}\right), \end{array}$$

where *p*_*t*_ is the model's estimated probability for the true class, *α*_*t*_ is a weighting factor for balancing the importance of positive/negative examples, and *γ* is a focusing parameter that adjusts the rate at which easy examples are downweighted [43]. In the RetinaNet implementation, typical values for *γ* are set to 2.0, which significantly downweights easy examples, as discussed in [44]. The parameter *p*_*t*_ is the probability output by the model for the positive class, which varies depending on the detection confidence. These parameters are tuned to enhance the detection accuracy and robustness of the model for detecting TB bacteria and WBCs.

#### 3.4.2. CNN Classifier

The CNN classifier employed softmax functions for prediction layers. Training utilized the SGD optimizer with a learning rate of 1–10^*−*3^ over 50 epochs. Pretrained networks, including VGG-16, ResNet-50, and LeNet-5, were initially trained on the CIFAR-10 dataset and then fine-tuned with our custom TB and WBC image patches. Additionally, smaller CNN architectures, such as AlexNet and SqueezeNet, were included for comparison.  Table 2 presents the metrics calculated from five runs of the Adam optimizer with a learning rate of 1–10^*−*3^. This comparison highlights the performance of the proposed classifier relative to both pretrained and smaller CNNs.

#### 3.4.3. Performance Metrics

Performance is evaluated using metrics such as average precision (AP) and mean average precision (mAP) to determine the effectiveness of the object detection model for TB bacteria. AP quantifies the precision of the model across various levels of recall and is computed as the area under the precision-recall curve for a given class [45]. mAP aggregates the AP scores across all classes to provide an overall assessment of the model's performance. It is defined as follows:

(2) $$\begin{array}{c} \text{mAP}=\frac{1}{N}\overset{N}{\underset{i=1}{\sum }}{\text{AP}}_{i}, \end{array}$$

where *N* represents the total number of classes and AP_*i*_ denotes the AP for class *i* [46].

Furthermore, additional performance metrics such as recall, precision, *F*1, and classification accuracy are employed to evaluate the classification model, as detailed in (3), (4), (5), and (6).

(3) $$\begin{array}{c} \text{Recall}=\frac{\text{TP}}{\text{TP}+\text{FN}}, \end{array}$$

(4) $$\begin{array}{c} \text{Precision}=\frac{\text{TP}}{\text{TP}+\text{FP}}, \end{array}$$

(5) $$\begin{array}{c} F1=\frac{2\times \text{Precision}\times \text{Recall}}{\text{Precision}+\text{Recall}}, \end{array}$$

(6) $$\begin{array}{c} \text{Classification accuracy}=\frac{\text{TP}}{\text{TP}+\text{FP}}, \end{array}$$

In these equations, TP, TN, FP, and FN refer to true positives, true negatives, false positives, and false negatives, respectively.

## 4. Results and Discussion

We evaluated the RetinaNet model's performance with various values of the *γ* parameter in the focal loss function to assess its effect on detection accuracy. The results for different *γ* values are summarized as follows in Table 2.

Table 2 presents the impact of adjusting *γ* on detection performance. Key findings include the following:

• *γ* = 0.5: The results show relatively lower AP for both WBCs and Bacilli, suggesting that this lower *γ* value does not adequately emphasize difficult-to-classify examples.
• *γ* = 1.0: There is a noticeable improvement in AP scores for both types of targets. This *γ* value introduces a balanced approach, enhancing model performance by addressing both easy and challenging examples.
• *γ* = 1.5: Further increases in AP and mAP indicate that this value provides a more effective balance, focusing more on harder examples while still performing well on simpler ones.
• *γ* = 2.0: This setting achieved the highest AP scores for both WBCs (0.93) and Bacilli (0.91), as well as the highest mAP (0.92). This optimal *γ* value significantly improves detection by emphasizing challenging examples.
• *γ* = 2.5: Although performance remains strong, a slight decrease in AP and mAP values compared to *γ* = 2.0 suggests that this higher *γ* might overly focus on difficult examples, slightly diminishing performance on easier cases.

These findings highlight that a *γ* value of 2.0 offers the best performance for both Bacilli and WBC detection, achieving the highest precision and mAP values. This suggests that a *γ* value of 2.0 is most effective for optimizing the focal loss parameter in the context of this study.

Our modifications to the ResNet-50 architecture can be extended to other deeper and wider residual networks, such as ResNet-101. Specifically, we replaced the Stride-2 convolutional layers in the initial blocks of Stages 4 and 5 of ResNet-50 with dilated convolutional layers, which preserve the spatial resolution of feature maps beyond Stage 4. ResNet-50 was chosen as the baseline model due to its balance between computational efficiency and accuracy, as more complex models generally improve mAP, though computational limits restrict our reporting to mAP improvements compared to the baseline. Anchors for region proposals were adjusted for the ResNet-50 model, with sizes set to cover the range of instance sizes in the dataset, as shown in Table 3.

**Figure 5:**
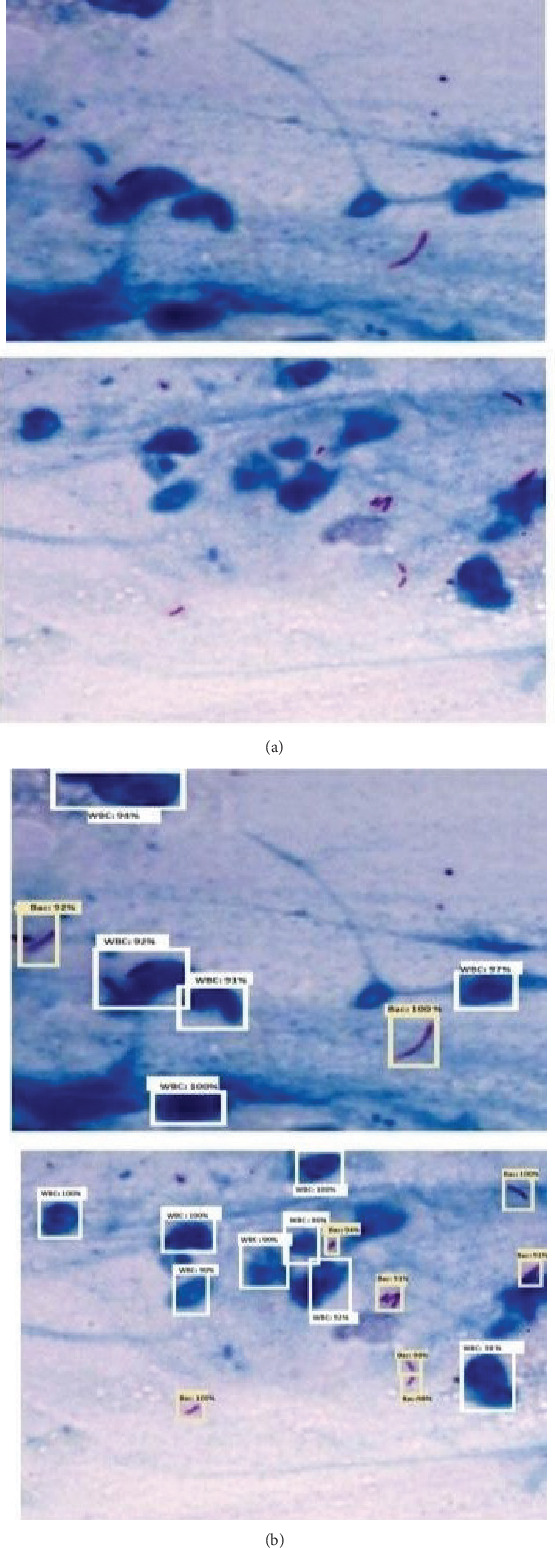
(a) The input image and (b) result obtained using RetinaNet with proposed backbone.

The proposed model performs ROI extraction from rectangular bounding boxes, with confidence values illustrated in Figure 5.

**Figure 6:**
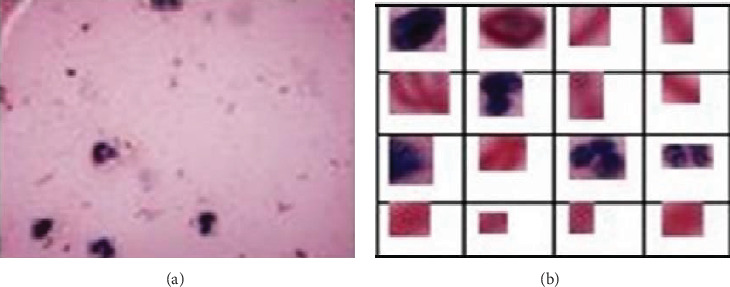
(a) Input image and (b) extracted ROIs.

ROI extraction was performed using anchor boxes, which are predefined bounding boxes with a range of sizes and aspect ratios. These anchor boxes act as reference points for detecting potential objects within the image. By systematically sliding these anchor boxes across different positions and scales within the image, regions likely to contain objects of interest are identified. The extracted ROIs are then subjected to further processing and analysis, as illustrated in Figure 6.

**Figure 7:**
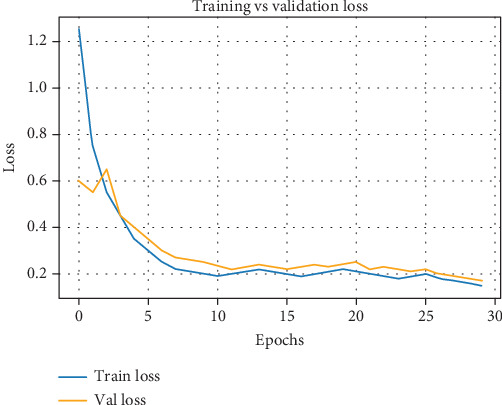
Training and validation loss curve of CNN-based image patch classifier.

Image patches with dimensions varying from 26 × 26 pixels to 128 × 128 pixels were extracted from the original images for input into the classification model. In total, 8000 patches were collected, including 3000 patches, each containing TB rod bacteria and WBCs used for training. The model was trained from scratch over 50 epochs. The resulting loss curve is presented in Figure 7. The proposed CNN classifier was fine-tuned using the Adam optimizer, testing various learning rates to analyze their impact on classification accuracy.  Table 4 summarizes the accuracy rates obtained with different learning rates after 50 training epochs. It is evident that a learning rate of 10^*−*4^ yielded the highest accuracy of 93%. Additional learning rates were tested to further examine their effect on model performance. As shown in the table, either too high or too low rates resulted in a noticeable decrease in accuracy, indicating the model's sensitivity to learning rate selection.

.

**Figure 8:**
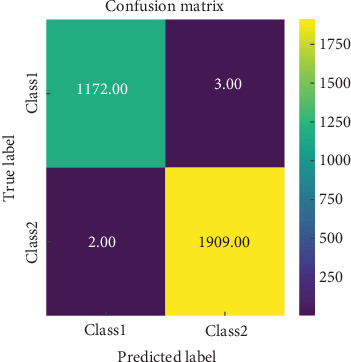
Confusion matrix of classification model.

A total of 1175 images of TB bacteria and 1911 images of WBCs were used to assess the classifier's accuracy thoroughly. Class-1 corresponds to TB bacteria in this setup, while Class-2 represents WBC. As illustrated in Figure 8, the confusion matrix shows that the classifier is highly effective at distinguishing between the two classes, with minimal false positives. This demonstrates the model's strong ability to accurately classify both TB bacteria and WBC, even in challenging image datasets. The model's overall precision and recall across both classes indicate its robustness, suggesting it can reliably perform in practical diagnostic scenarios.

Then, 8000 images containing both TB bacteria and WBCs were used to train the classifier model. To evaluate the performance of the proposed classifier, we calculated metrics such as recall, precision, *F*1 score, and classification accuracy. These metrics are summarized in Table 5.

The table compares several classifiers based on four critical metrics: precision, recall, *F*1 score, and accuracy. Among these, the proposed CNN demonstrates the highest performance, with a precision of 92.5%, recall of 91.5%, *F*1 score of 91.9%, and an accuracy of 93%. This signifies the proposed CNN's superior ability to detect and classify instances compared to other models accurately.

EfficientNet-B0 follows closely, delivering strong results with an *F*1 score of 91.2% and accuracy of 92%, though it falls marginally short of the proposed CNN in terms of precision and *F*1 score. Xception, with a precision of 92% and accuracy of 92%, offers competitive performance but lags slightly in recall. InceptionV3, with balanced metrics (precision: 91%; recall: 90%; and accuracy: 92%), shows commendable performance but still does not surpass the proposed CNN.

DenseNet-121 presents solid metrics (precision: 90%; recall: 89.5%; and accuracy: 91%) but performs slightly below the top models. Older architectures like AlexNet (precision: 88% and accuracy: 89%) and SqueezeNet, known for its parameter efficiency (precision: 86% and accuracy: 87%), show decent performance but are less effective than modern classifiers. Similarly, ResNet-50 delivers strong results (precision: 87% and accuracy: 88%) but is outperformed by newer models.

MobileNetV2 offers a balance between efficiency and performance, with metrics close to the top models (precision: 89.5% and accuracy: 90%). VGG-16, a well-known model, delivers robust performance (precision: 89% and accuracy: 90%), yet does not outperform the proposed CNN. Lastly, LeNet-5, as an early CNN model, shows the lowest performance with an *F*1 score of 82.5% and accuracy of 83%, highlighting its limitations compared to more advanced architectures.

Overall, this comparison underscores the advanced performance of the proposed CNN, demonstrating its superiority in classification tasks while offering insight into the strengths and weaknesses of other models. The results highlight the effectiveness of the proposed CNN model, which achieves higher precision in distinguishing between TB bacteria and WBCs compared to the other classifiers.

The comparison table illustrates that the Proposed CNN achieves the highest performance across all metrics, with a precision of 92.5%, recall of 91.5%, *F*1 score of 91.9%, and accuracy of 93%. This clearly demonstrates the superiority of the proposed CNN in classification tasks, outperforming other state-of-the-art (SOA) models like EfficientNet-B0, Xception, InceptionV3, and DenseNet-121.

In addition to performance metrics, it is important to assess how efficiently these models utilize computational resources, particularly when considering real-time applications. The proposed CNN not only achieves top-tier performance but also designed to be lightweight, requiring less memory and computation time. The following table highlights the model's space and time utilization compared to other SOA models.

The ablation study of the proposed CNN model, as shown in Table 6, highlights the importance of key architectural components. The full model achieves the highest performance, demonstrating its effectiveness. Removing batch normalization results in a notable performance drop, underscoring its role in stabilizing and improving model training. Excluding dropout slightly decreases performance, emphasizing the significance of regularization in preventing overfitting. Reducing the number of convolutional layers significantly lowers accuracy, indicating the need for deeper feature extraction. Similarly, using smaller filter sizes degrades performance, suggesting that larger filters capture more meaningful features. Lastly, removing data augmentation reduces the model's robustness, limiting its exposure to diverse training data and affecting generalization.

**Figure 9:**
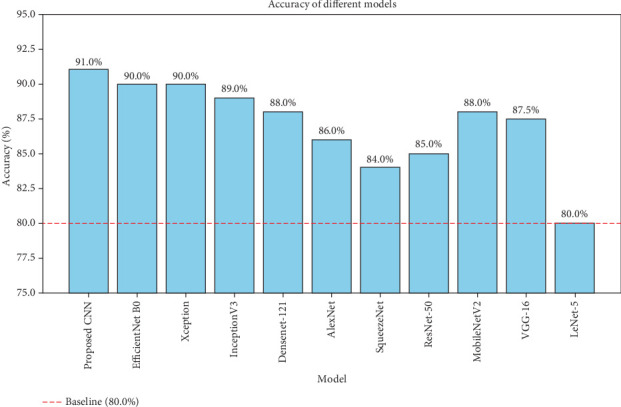
Test accuracy of the different models.

After training, a set of 3000 test images was used to evaluate the final accuracy of the classifiers. As shown in Figure 9, the proposed CNN model performed the best, with a classification accuracy of 91%, whereas AlexNet and SqueezeNet achieved accuracies of 86% and 84%, respectively.

Feature maps throughout the CNN model were examined as shown in Table 7, focusing on specific layers selected during the training phase. These visualizations provide valuable insights into the model's capability to emphasize pertinent regions within the images.

Figure 8 displays the confusion matrix for the classification of test images from TB and WBC categories. The matrix demonstrates that the proposed model effectively distinguishes between the two classes, achieving high classification accuracy with minimal occurrence of false positives.

Figure 9 presents a detailed view of the classification results for a TB test image.

This figure highlights the model's high confidence in identifying key regions of interest. The visualized feature maps reveal how the CNN model captures and processes relevant features, underscoring its proficiency in recognizing and classifying specific image characteristics associated with TB. The results indicate that the model's performance is robust, with a strong emphasis on classification accuracy and a low misclassification rate.

**Figure 10:**
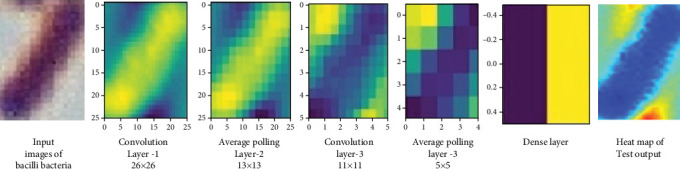
Displays the visualization of different layers within the proposed classifier, encompassing Convolution Layer 1, Average Pooling Layer 1, Convolution Layer 2, Average Pooling Layer 2, dense layer, and the heat map corresponding to the test input image.

Figure 10 Illustrates the different layers of the proposed classifier, including Convolution Layer 1, Average Pooling Layer 1, Convolution Layer 2, Average Pooling Layer 2, the dense layer, and the heat map for the test input image. The two-stage approach we propose is highly effective for detecting bacteria, especially given the challenges posed by their small size, sparse distribution, and often low-contrast appearance in complex backgrounds.

In many microscopy or culture plate images, bacterial colonies are tiny and difficult to distinguish, which limits the effectiveness of conventional object detection models. By using RetinaNet with a FPN and focal loss, our method can better detect these small and unevenly distributed targets, improving sensitivity and reducing false negatives. The integration of dilated convolutions in the feature pyramid helps capture broader context without losing spatial detail, which is crucial when trying to separate bacterial colonies from background noise. The second stage of the pipeline, a focused patch-based CNN, further improves classification by examining each detected region more closely, allowing for accurate identification of colony types or patterns. This flexible, modular design makes the system well-suited for various imaging conditions, offering high accuracy and reliability in medical or laboratory-based bacterial detection tasks. The overall explanation is given in Table 8 given below.

## 5. Conclusion

In summary, the detection and classification of TB bacteria, given their diminutive size and frequent overlap with WBCs, pose significant challenges in clinical diagnostics. This study has introduced an advanced classification model tailored to differentiate between TB bacteria and WBCs within small image patches. The model's performance has been rigorously evaluated against established architectures, including VGG-16, ResNet50, LeNet-5, AlexNet, and SqueezeNet.

Significantly, the proposed classifier demonstrated a commendable accuracy of 93%, underscoring its efficacy in accurately identifying TB bacteria amidst challenging conditions. Further enhancement in the model's performance was achieved by incorporating focal loss within the RetinaNet framework. Focal loss, designed to address class imbalance by focusing more on hard-to-classify examples, proved instrumental in improving the detection accuracy of rare TB bacteria. The integration of RetinaNet, with its capability to handle varying object scales and its refined approach to bounding box regression, further contributed to the classifier's superior performance.

Overall, the combination of the novel classification approach with advanced loss functions and detection frameworks has resulted in a robust and highly accurate system for TB bacteria detection, showcasing a significant advancement in the realm of medical image analysis.

## Figures and Tables

**Table 1:** Convolutional neural network model architecture for tuberculosis bacteria detection.

**Layer**	**Input size**	**Output size**	**Details**
Conv + ReLU	26 × 26 × 3	13 × 13 × 16	16 filters, 3 × 3, ReLU activation
Maxpool + ReLU	13 × 13 × 16	11 × 11 × 32	2 × 2 pool size, ReLU activation
Conv + ReLU	11 × 11 × 32	9 × 9 × 32	32 filters, 3 × 3, ReLU activation
Maxpool + ReLU	9 × 9 × 32	7 × 7 × 64	2 × 2 pool size, ReLU activation
Conv + ReLU	7 × 7 × 64	5 × 5 × 128	128 filters, 3 × 3, ReLU activation
Maxpool + ReLU	5 × 5 × 128	Flattened	2 × 2 pool size, ReLU activation
Dropout	Flattened	Flattened	Dropout rate 0.5
Dense + ReLU	Flattened	1 × 1 × 1024	Fully connected, ReLU activation
Dropout	1 × 1 × 1024	1 × 1 × 1024	Dropout rate 0.5
Dense + ReLU	1 × 1 × 1024	1 × 1 × 128	Fully connected, ReLU activation
Output (Softmax)	1 × 1 × 128	Classification	Softmax activation for classification

**Table 2:** Performance metrics of RetinaNet with different *γ* values in focal loss.

**γ**	**AP-WBC**	**AP-Bacilli**	**mAP**
0.5	0.88	0.85	0.86
1.0	0.90	0.87	0.88
1.5	0.92	0.89	0.90
**2.0**	**0.93**	**0.91**	**0.92**
2.5	0.92	0.90	0.91

*Note:* The bold values indicate that the analysis shows that the choice of *γ* in focal loss plays a crucial role in balancing detection performance. Notably, *γ* = 2.0 delivers the highest precision for both WBC (0.93) and Bacilli (0.91), along with the best overall mAP (0.92). This demonstrates that *γ* = 2.0 optimally balances easy and hard examples during training, thereby enhancing RetinaNet's robustness and generalization ability for medical image detection.

**Table 3:** Performance comparison of RetinaNet with various backbones.

**Backbone**	**Dataset (train/test)**	**AP-WBC**	**AP-Bacilli**	**mAP**
ResNet-34	Training set Test set	0.89 0.80	0.87 0.83	0.85 0.81
ResNet-50	Training set Test set	0.93 0.84	0.91 0.87	0.89 0.82
ResNet-101	Training set Test set	0.92 0.80	0.89 0.82	0.86 0.81
ResNet-152	Training set Test set	0.91 0.82	0.90 0.83	0.87 0.80
**Proposed backbone**	**Training set** **Test set**	**0.92** **0.81**	**0.95** **0.86**	**0.92** **0.89**

*Note:* The bold values indicate that the proposed backbone achieved the best results, with a training mAP of 0.92 and a test mAP of 0.89, outperforming all ResNet-based backbones.

**Table 4:** Performance of CNN classifier with different learning rates using Adam optimizer over 50 epochs. The results highlight the best learning rate, yielding the highest accuracy, with a comparative analysis of other rates.

**Learning rate**	**Accuracy rate**
0.01	89%
0.001	90%
**0.0001**	**93%**
0.00001	91%
0.1	85%
0.00005	87%
0.000001	88%

*Note:* The bold values indicate that at a learning rate of 0.0001, the CNN achieved the highest accuracy (93%), indicating it as the optimal choice compared to both higher (unstable) and lower (slower) rates.

**Table 5:** Performance comparison of different classifiers.

**Classifier**	**Precision (%)**	**Recall (%)**	**F**1** score (%)**	**Accuracy (%)**
Proposed CNN	92.5	91.5	91.9	93
EfficientNet-B0	91.5	91.0	91.2	92
Xception	92.0	91.0	91.5	92
InceptionV3	91.0	90.0	90.5	92
DenseNet-121	90.0	89.5	89.7	91
AlexNet	88.0	87.0	87.5	89
SqueezeNet	86.0	85.0	85.5	87
ResNet-50	87.0	86.0	86.5	88
MobileNetV2	89.5	89.0	89.2	90
VGG-16	89.0	88.5	88.7	90
LeNet-5	82.0	83.0	82.5	83

**Table 6:** Comparison of model utilization: memory, parameters, and inference time.

**Classifier**	**Model size (MB)**	**Parameters (millions)**	**Inference time (ms)**
Proposed CNN	25	12.0	15
EfficientNet-B0	29	5.3	25
Xception	88	22.9	30
InceptionV3	92	23.9	35
DenseNet-121	33	8.0	27
AlexNet	240	61.0	45
SqueezeNet	5	1.2	20
ResNet-50	98	25.6	40
MobileNetV2	14	3.4	22
VGG-16	528	138.0	50
LeNet-5	1	0.06	10

**Table 7:** Ablation study of the proposed CNN model.

**Configuration**	**Precision (%)**	**Recall (%)**	**F**1** score (%)**	**Accuracy (%)**
Full model (proposed CNN)	92.5	91.5	91.9	93
Without batch normalization	89.0	88.5	88.7	90
Without dropout	90.5	89.0	89.7	91
Reduced convolution layers	87.0	85.0	86.0	88
Smaller filter size	85.5	84.0	84.7	87
Without data augmentation	88.0	87.5	87.7	89

**Table 8:** : Comparison of YOLO, faster R-CNN, and the proposed RetinaNet + patch-based CNN method for detecting small and fine-grained objects. The proposed approach shows superior accuracy and adaptability for tasks like gender and bacterial classification.

**Criteria**	**YOLO (v3–v8)**	**Faster R-CNN**	**Proposed method (RetinaNet + patch-based CNN)**
Detection type	One-stage	Two-stage	Two-stage (RetinaNet + custom CNN)
Speed (inference)	Very High (Real-time)	Moderate to Low	Moderate (RetinaNet) + lightweight CNN (Balanced)
Accuracy (mAP)	High for large objects, moderate for small	Very High across object sizes	High, especially for small/fine-grained objects
Small object detection	Moderate (misses fine details)	Good, but dependent on RPN	Excellent (FPN + dilated conv + patch refinement)
Handling of class imbalance	No built-in solution	Basic softmax, often needs weighted loss	Focal loss (in RetinaNet) effectively handles class imbalance
Feature aggregation	Single scale	Multi-scale (limited FPN)	Enhanced FPN + L2 Normalization + Dilated Blocks
Modularity/customization	Low	Medium	High (Modular two-stage with task-specific CNN)
Best use case	Real-time applications (e.g., surveillance)	High-accuracy needs (e.g., medical imaging)	Fine-grained tasks (e.g., poultry gender classification)
Complexity of implementation	Low	High	Medium (modular and scalable)
Performance on your dataset	Moderate precision and recall	High precision, moderate recall	Highest

## Data Availability

The datasets generated during and/or analyzed during the current study may be available from the corresponding author upon reasonable request.
